# Continuous flow nitration in miniaturized devices

**DOI:** 10.3762/bjoc.10.38

**Published:** 2014-02-14

**Authors:** Amol A Kulkarni

**Affiliations:** 1 Chem. Eng. & Proc. Dev. Division, CSIR-National Chemical Laboratory, Pune – 411 008, India, phone: +91-20-25902153

**Keywords:** continuous flow, flow chemistry, nitration, nitric acid, microreactors, tubular reactor

## Abstract

This review highlights the state of the art in the field of continuous flow nitration with miniaturized devices. Although nitration has been one of the oldest and most important unit reactions, the advent of miniaturized devices has paved the way for new opportunities to reconsider the conventional approach for exothermic and selectivity sensitive nitration reactions. Four different approaches to flow nitration with microreactors are presented herein and discussed in view of their advantages, limitations and applicability of the information towards scale-up. Selected recent patents that disclose scale-up methodologies for continuous flow nitration are also briefly reviewed.

## Review

### 1 Introduction

Nitration of aromatics is one of the oldest and industrially most important reactions. A reaction between an organic compound and a nitrating agent leads to the introduction of a nitro group onto a carbon, nitrogen or oxygen atom of that organic compound [1]. Among the earliest reports are those of Faraday nitrating benzene, the synthesis of nitrobenzene by Mitscherlich [2] using benzene and fuming nitric acid, and the use of mixed acids (nitric acid and sulfuric acid) for aromatic nitration by Mansfield [3]. In general, nitration reactions are fast and highly exothermic. Typically, the nitration of aromatic compounds is acid-catalyzed and it involves an electrophilic substitution where the nitronium ion (NO_2_^+^) acts as the reactive species [4–6].

Based on estimations of 2007 and the proposed world production capacity, the overall world production of nitric acid in 2012 is assumed to be close to 78 Mi TPA, of which 85% is used for the production of ammonium nitrate as fertilizer and 6% for production of nylon. The remaining 9% – that is about 8 Mi TPA – are used for the nitration of aromatics [7]. Nitro derivatives of aromatic compounds are used in variety of basic chemicals, specialty chemicals, and knowledge chemicals. They are also employed in dyes, perfumes, pharmaceuticals, explosives [8], intermediates, colorants, and pesticides. In general, the annual demand for nitric acid grows in the range of 3 to 6%. A large proportion of nitric acid consumed during aromatic nitration is directed towards the synthesis of aniline derivatives, which are produced by nitration followed by reduction. These aniline derivatives find applications in insecticides, pigments, dyes, resins, textiles, elastomers, plant growth regulators, pharmaceuticals, fuel additives, antioxidants, and rubber accelerators. In the leather industry *m*-nitrophenol is used as a fungicide and *p*-nitrophenol as a chemical intermediate for leather preservatives. 2,4-Dinitrophenol is useful for the manufacturing of photographic developers and serves as a wood preservative and also as an insecticide. 4-Amino-2-nitrophenol and 2-nitro-*p*-phenylenediamine are components of permanent hair dye products and fur dye. Several aniline derivatives are also used for the synthesis of various dyes [9], the first one being the aniline Yellow [10] reported in 1880. The history of the relevance of nitration for the dye and colorant industry covers more than a century. Nitro derivatives of the toluene diisocynate are employed in the manufacturing of flexible polyurethane foams, which are used in transformation, furniture, and carpet underlay. 2,4,6-Trinitrotoluene is a military and industrial explosive. Nitro derivatives of glycerine, urea and naphthalene also exhibit explosive properties. Some of the aniline based dyes were used for medical applications, too. The extension of the aniline based medicines led to hundreds of drugs [11], which were used for medication during and after World War II (1939–45). Several nitro derivatives are applied to the synthesis of the respective amino groups, which form important building blocks in the synthesis of active pharmaceutical ingredients (APIs). Almost 65% of APIs requires at least one nitration step in the whole process. Among the other basic chemicals that are used in significantly large quantities are a large number of organic molecules, e.g., nitrobenzenes, nitrophenols, nitrotoluenes, nitroxylenes, nitronaphthalenes, nitrohaloaromatics, nitroanilines, nitrotoluidines, imidazole derivatives, nitroketones, pyridine and quinoline derivatives, and nitro alcohols. Thus, part of the human life and life style is dependent of nitration as a unit reaction.

**Figure 1:**
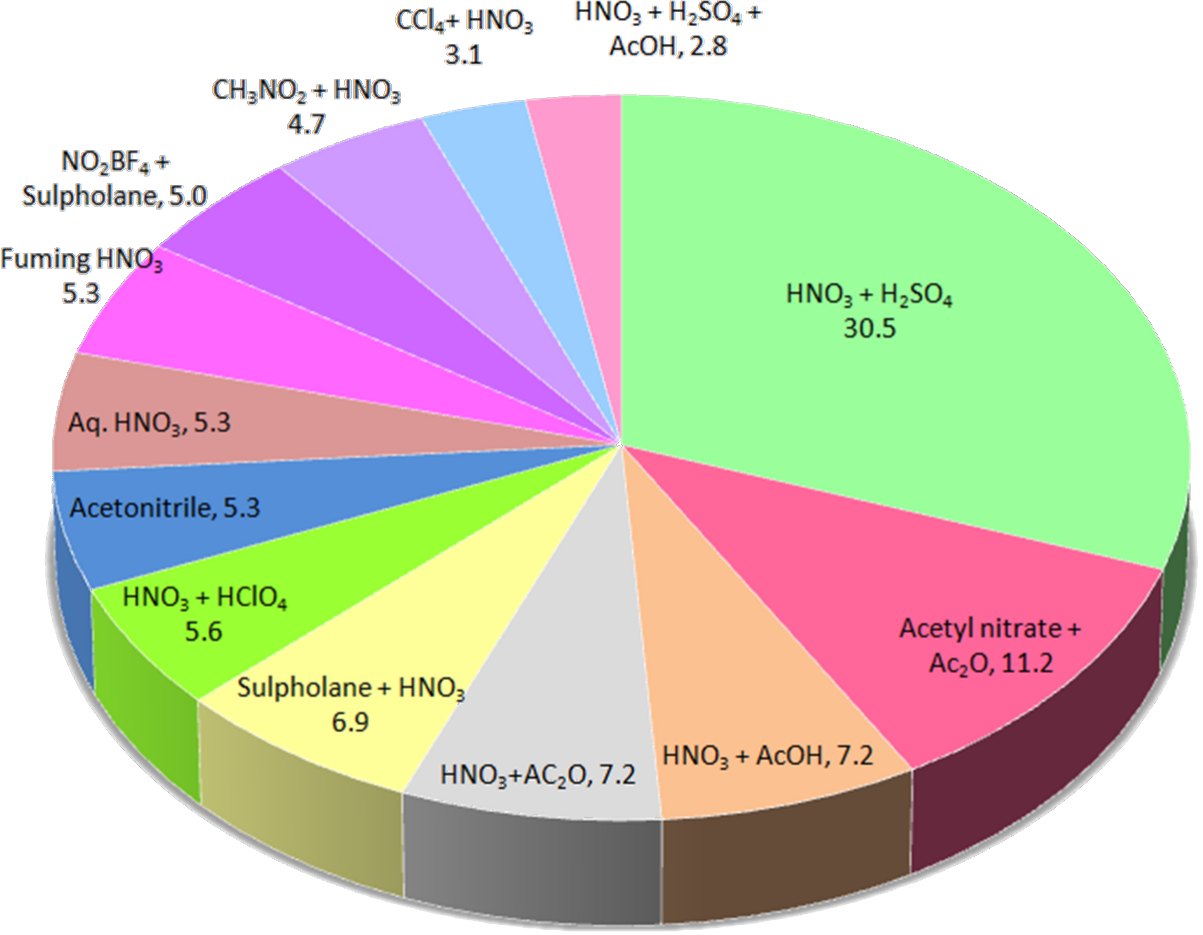
Analysis of the literature on aromatic nitration over the last 50 years. Numbers next to each nitrating agent correspond to the percentage of literature in which the respective agent is used for nitration.

In general, several types of nitrating agents are used for nitration. A literature search covering the last 50 years is presented in Figure 1. One third reports on nitrations of organic substrates with sulfuric acid and nitric acid as the nitrating agents. However, the use of other activating agents (e.g., acetic anhydride) is not uncommon. Typically, nitrations with undiluted nitric acid generate water that leads to the dilution of the nitric acid, so that the concentration of the nitronium ions and thus the reaction rates are reduced. It also gives a lower selectivity due to the oxidation of the aromatic substrate. The isolation of the product from the organic phase is problematic, even after complete conversion is achieved. Nitric acid is commonly used in excess and it can form a complex with the organic products, so that only after reaction, significant dilution with water allows separating the organic phase from the diluted nitric acid. The sulfuric acid in so-called “mixed acid”, i.e., a mixture of HNO_3_ and H_2_SO_4_, catalyzes the generation of nitronium ions and extracts water, which is generated from the dissociated nitric acid. Usually, the sulfuric acid is used in excess in the preparation of the mixed acid. Therefore, in the presence of sulfuric acid nitrations are usually faster and selective. A variety of other acids including solid acids can be used in place of sulfuric acid to enhance the rates of nitration. This review focuses on continuous nitration under flow conditions, while the mechanisms of nitration will not be discussed in detail, as they are well understood [12]. In the absence of any other acid, nitric acid alone can act as a self-protonating agent or self-catalyst in which one molecule of nitric acid protonates a second one leading to the formation of a nitronium ion [13]. The electrophilic nitration is the most common reaction. It proceeds through the nitronium ion NO_2_^+^ as an electrophilic species. One of the most widely accepted mechanism for the electrophilic nitration involves the sequence of reactions depicted below [12]:

[1]
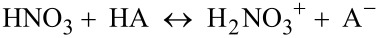


[2]
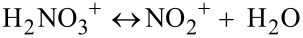


[3]
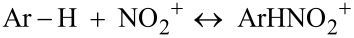


[4]
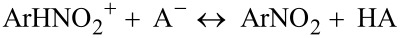


In the first step, the presence of a strong acid catalyst HA protonates HNO_3_ thereby releasing the reactive species NO_2_^+^ and water in the second step. Both of these steps are rapid and reversible. The third step is much slower – and hence rate-controlling – than the reverse of the second step due of the presence of water and the poor solubility of the aromatic species in the mineral acid medium. In the rate-controlling step the nitronium ion attacks the aromatic ring to give an intermediate carbocation, which deprotonates rapidly to afford the nitroaromatic product in the final step. Therefore, nitration in mineral acid exhibits a second-order kinetic behavior, first-order in HNO_3_ as well as in the aromatic substrate. However, the reaction rates strongly depend upon the strengths of the cumulative acid [14]. The strength of the nitric acid and the quantity of the sulfuric acid necessary for a given nitration depend on the substrate and the desired extent of nitration. The weight ratio of consumed sulfuric acid to the weight of water in the final acid (spent acid) after the nitration is complete is termed the dehydrating value of sulfuric acid (D.V.S.). The D.V.S. value is estimated by multiplying the mol ratio of sulfuric acid to water with 5.444. This is one of the most common parameters for exploring the nature of a substrate that undergoes nitration [15]. However, the nature of nitration and the corresponding heat effects largely depend upon the nitrating agent, the conditions of the nitration, and the reactivity of the aromatic substrate [16].

Generally, the economics of a nitration process largely depends on the procedure used to remove the water from the system and the regeneration of the dehydrating agent [17]. Theoretically, the application of undiluted nitric acid – usually used in excess – is expected to avoid the use of water. In practice, however, the separation of nitroarenes is facilitated in the diluted nitric acid. Nitration with diluted nitric acid generates the nitronium ion in the following manner:

[5]
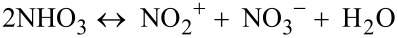


In general, this approach is not considered economically feasible, so that most nitrations are carried out with the next cheapest nitrating agent i.e., a mixture of nitric and sulfuric acids. If they are used in excess, both of them are usually treated by neutralization, by feeding the weak nitric acid to an absorption tower, or recovered from the spent acid. Generally, the latter two options are not viable unless the quantity is very large. A simple neutralization is unacceptable given stringent environmental regulations. Adherence to regulations and requirements adds to the process costs [17].

### 2 Nitration: modes and systems

Conventional aromatic nitration usually follows a batch or a semi-batch approach, where the mixing of reactants and the reaction itself are carried out very slowly [18–19]. Some of the most important concerns, which do not allow for an easy scale-up include: (i) an inadequate heat transfer area (20–100 m^2^/m^3^), (ii) an inhomogeneous system, mainly due to immiscible substrates and inefficient mixing, leading to mass transfer limitations, (iii) batch to batch variation in the degree of conversion, yield and selectivity, (iv) prolonged reaction times, (v) reactions at very low temperatures to reduce the rate of heat generation, (vi) the use of excess nitrating agent, mainly the spent acid, which occupies significant volume, has to be neutralized thereby needing large quantity of water, and generates inorganic salts. As one or more of these limitations are experienced in every batch operation it is necessary to check their feasibility for continuous flow processing. Such transformations from batch to flow have been carried out for a lot of reactions and many continuous nitration plants of commercial scale exist. However, the real challenge is in taking-up such an exercise for products where these transformations will not only help to overcome safety issues, but also significantly enhance the yield of the desired isomer. Having the importance of nitration in mind and considering the challenges industrial nitration is faced with in terms of the sustainability of individual processes a few large scale consortia have focused on continuous flow nitration using miniaturized devices [20–21].

An analysis of the literature shows that as many as 45% of the nitrations are for liquid phase systems both homogeneous (miscible) and heterogeneous (immiscible) with only 24% for homogeneous systems. Most of the remaining examples involve the substrate in solid phase. Only very rarely is the nitration of gases reported in the literature. Thus, suitable devices and equipment for nitrations are determined by the phases involved and their activity. The presence of multiple phases clearly indicates the choice of substrates explored in the nitrations reported so far and the use of solvents for specific substrates, mainly to maintain the system in liquid phase (although immiscible). More details on the selection of the experimental setup will be elaborated in section 3.

In this review, we analyze recent studies on continuous flow nitration using miniaturized flow reactors. We provide a guideline that helps to quickly decide under which conditions it is worthwhile to conduct continuous flow nitration from a practical point of view. Key features of this report are: (a) a thorough overview on continuous flow nitrations, (b) a discussion on general issues that have to be considered when conducting continuous nitrations, (c) how data from individual reactions are collected and analyzed in order to devise scale-up or numbering-up processes or extend the approach for the continuous preparation of other derivatives and (d) guidelines supporting to identify the best setup for continuous flow nitrations using microreactors.

### 3 Continuous flow nitration

During World War II, both batch and continuous flow nitration were conducted for the production of different nitroarenes (e.g. nitroglycerin, ethylene glycol dinitrate, diethylene glycol dinitrate, cyclotrimethylenetrinitramine, pentaerythritol tetranitrate, nitrocellulose, etc.). Continuous processes were enforced as they allowed to retain the same scale of operation while keeping the plant size limited [17]. Continuous apparatus for the nitration of solid materials and the production of solid nitrated compounds were commercially used in several European countries. Yet, as of today, a large number of nitrations are still conducted in batch mode across the world. The primary reasons for the batch mode approach are the small scale and infrequent production owing to multipurpose facilities. However, even these small production centers leave a large chemical footprint. Therefore, many examples of efficient continuous flow nitrations have been established in the last few years.

The initial development and demonstration of the continuous flow synthesis using miniaturized devices or microreactors took place in academia and research institutions. The benefits of the approach are particularly evident for highly exothermic reactions and for reactions involving unstable intermediates. Later, this approach was adopted by the industry and feasibility studies on microreactors provided concepts for pilot plant development and commercial scale manufacturing. Early explorations included the nitration of aromatic substrates. Selected references on continuous flow nitration are given in Table 1. In the following, we analyze and discuss important points collected from the literature, which are relevant for the experimental setup, the scale of operation, the reproducibility, the lack of data etc. This examination may be helpful to decide whether the current level of knowledge is sufficient to extend the approach of continuous flow nitration to other aromatic substrates and whether the available data are sufficient to for a scale-up.

#### 3.1 Analysis of the literature

**Figure 2:**
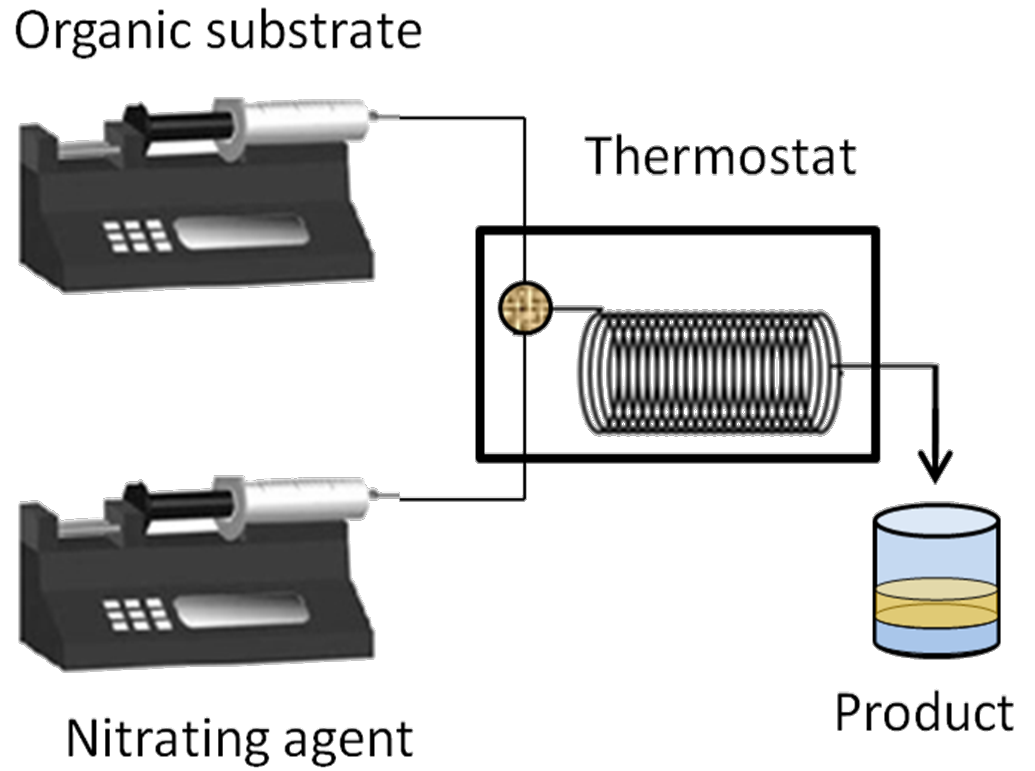
Schematic of a typical experimental setup for aromatic nitration. The circular segment shown inside the thermostat is usually a micromixer.

The literature that covers continuous flow nitration can be coarsely classified on the basis of (a) the nitrating agent, (b) the type of reaction device, (c) the property of the system being homogeneous or multiphase, and (d) the exothermic extent of individual reactions. A typical experimental setup for continuous flow nitration includes pumps for the dosing of reactants, a micromixer for the rapid and efficient mixing of these reactants, and a residence time unit, which may be either a microfluidic device with channels or a tube.The residence time unit is either immersed in a constant temperature bath or has built-in cooling/heating systems to maintain a specific temperature. A schematic of such a setup is shown in Figure 2. In Table 1 specific parameters are shown to provide a firsthand overview of the typical conditions and setups. In general, the heat of reaction for all of them ranges from −73 to −253 kJ/mol, and almost all substrates are in liquid phase, propane being the sole exception. Typical residence times are between 5 s to 15 min corresponding to an average heat release rate of −10 to −50 kJ/s/mol. These numbers require a heat transfer area per unit volume between 300 and 2540 m^2^/m^3^, which corresponds to channel diameters in the range of 0.0016 to 0.013 m. These dimensions are actually close to the channel diameters used in most of the experiments. In this review, we have classified the literature on the basis of the nitrating agent, so that the comments on hydrolytic termination, isolation and other work-up aspects can be separately applied for each class.

**Table 1:** Summarized literature survey on continuous flow nitration using microreactors (NA: nitric acid, Org: organic substrate).

No.	Author (year)	Substrate	Experimental conditions			% Conversion [% selectivity] (% yield)
			Temp. (°C)	τ (min)	Nitrating agent	

1	Denton et al. [22]	2-nitropropane	204–232	10	70% HNO_3_ (NA/Org = 1)	50
2	Veretennikov et al. [23]	chlorobenzene	65	10	97% HNO_3_ (NA/Org = 3)	99
3	Anderson [24]	2-amino-6-chloro-4-hydroxy- 1,3-diazine	45	2.5	90% HNO_3_ (Org + H_2_SO_4_)	85
4	De Jong et al. [25]	2-amino-4-chloro-6-hydroxy- pyrimidine (+ H_2_SO_4_)	45	7 h	90% HNO_3_ (NA:Org = 3.09:1)	81.60
5	Dagade et al. [26]	toluene	120	–	NA:Org = 0.59:1	55 [73]
6	Panke et al. [27]	pyrazole-5-carboxylic acid (+ H_2_SO_4_)	90	35	HNO_3_ + H_2_SO_4_	(73)
		2-methylindole (+ H_2_SO_4_)	3	0.8	NaNO_3_ + H_2_SO_4_	(70)
		pyridine *N*-oxide (+ H_2_SO_4_)	120	78	Nitrating mixture	(78)
		toluene (+ Ac_2_O + H_2_SO_4_)	30	70	neat HNO_3_	–
7	Antes et al. [28]	toluene	−10	3 s	NA:Org = 2.56:1	89–92
8	Ducry and Roberge [29]	phenol	45		NA:Org = 1.8:1 (Org:AcOH = 1:6)	75 [79.4] (o:m ~ 1.1)
			20	15	NA:Org = 1.4:1	77 [74.6] (o:m ~ 1)
9	Kulkarni et al. [30]	salicylic acid	50	7	HNO_3_ + AcOH (AcOH:Org = 10)	100
10	Pelleter & Renaud [31]	3-methylpyrazole (+ H_2_SO_4_)	65	90	69% HNO_3_ (NA:Org = 13)	[88]
		3-ethyl-1*H*-pyrazole (+ H_2_SO_4_)	65	25	69% HNO_3_ (NA:Org = 33)	[55]
11	Yang et al. [32]	benzene	75	72 s	NA:Org = 3.1:1	44.70 [99.9]
12	Kockmann & Roberge [33]	phenol	20		65% HNO_3_ (NA:Org = 1.4:1)	77 [74.6]
13	Shen et al. [34]	isooctanol	35	7.2 s	Nitrating mixture (NA:Org = 1.5:1)	98.20
14	Kulkarni et al. [35]	benzaldehyde	5		Nitrating mixture (NA:Org = 3.5:1)	100
15	Brocklehurts et al. [36]	8-bromo-1*H*-quinolin-2-one	90	3	100 % HNO_3_ (NA:Org = 20:1)	100
		1-benzosuberone	10	5	(NA:Org = 10:1)	79
16	Knapkiewicz et al. [37]	2-isopropoxybenzaldehyde	10	5.4 s	red fuming HNO_3_ (NA:Org = 6.47:1) (Org + dichloromethane)	65
17	Löwe et al. [38]	propane	385–455	1 s	(NA/Org = 1)	2
18	Gage et al. [39]	*N*-(5-bromo-4-methylpyridine- 2-yl)acetamide	0–5	11 h	Fuming HNO_3_ + H_2_SO_4_ (NA/Org = 1.1) (Org+SA)	50 [99]
19	Yu et al. [40]	*p*-difluorobenzene	30–70	0.3–1	Nitrating mixture (SA/NA = 1.8)	98 [99]
20	Chen et al. [41]	*N*-(1-ethylpropyl)-3,4-xylidine	60–90	0.8–9 s	HNO_3_ 65–98% (NA/Org = 4.3)	100 [92–99]
21	Burns and Ramshaw [42]	benzene and toluene	25 to 60			

**3.1.1 Nitration with mixed acids.** As mentioned above 30% of reported nitrations utilize mixed acids as a nitrating agent. The nature of mixed acids varies from system to system. In one of the first feasibility studies under continuous flow conditions Burns and Ramshaw [42] used a simple T-junction with three intersecting channels followed by a coiled capillary (length ~30 cm and 180 cm, i.d. ~0.127 mm to 0.3 mm), which served as a microreactor system. The reactor coil was placed on a hot plate that was insulated with layers of polyurethane foam to ensure a minimum heat loss. The experimental setup resembles the schematic shown in Figure 2. It was observed that the concentration of nitrobenzene increases with reactor length at different concentrations of the sulfuric acid in nitric acid. The initial nitration rates were governed by the kinetics and by mass transfer limitations. Both of these regimes strongly depend on the concentration of sulfuric acid given at a fixed wt % of nitric acid. In case of toluene nitration, at a given fixed composition of the nitrating agent the initial rate doubles with an increase in the reactor temperature from 25 °C to 60 °C over a wider range of inlet flow ratio of the two phases. Interestingly, the initial reaction rates are reported to be higher when the inlet velocity of the reacting mixture is increased, a fact later elaborated in detail by Dummann et al. [43].

**Scheme 1:**
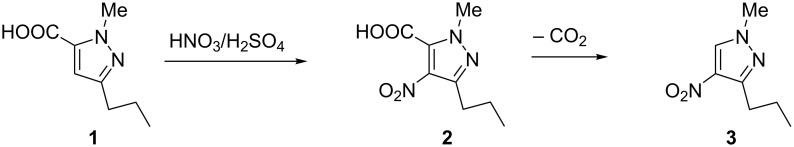
Nitration of substituted pyrazole-5-carboxylic acid **1**. *T* = 90 °C, residence time = 35 min, yield: 73% [27].

**Scheme 2:**
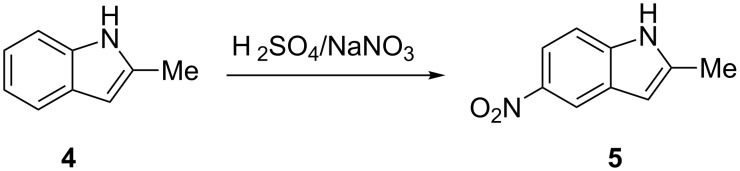
Nitration of 2-methylindole (**4**). *T* = 3 °C, residence time = 48 s, yield: 70%. [27].

**Scheme 3:**
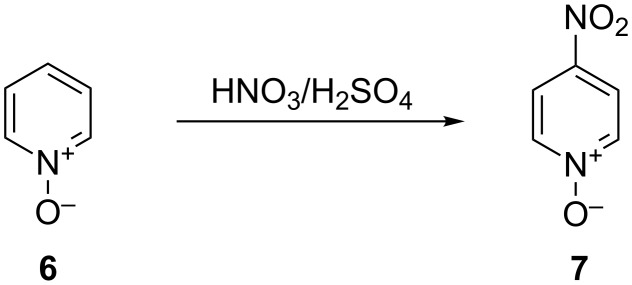
Nitration of pyridine-*N*-oxide (**6**), *T* = 120 °C, residence time = 80 min, yield: 78% (72% in the flask experiment) [27].

**Scheme 4:**
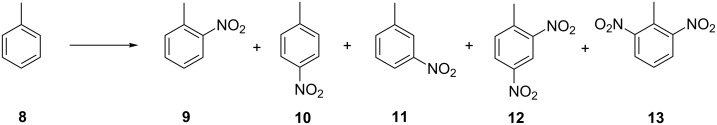
Nitration of toluene (**8**). Method 1: H_2_SO_4_/HNO_3_, *T* = 65 °C, residence time = 15 min. Method 2: Ac_2_O/H_2_SO_4_/HNO_3_, *T* = 30 °C, residence time = 70 min (higher selectivity and major products 2-nitrotoluene (**9**) and 4-nitrotoluene (**10**) were obtained in 54% and 39%, respectively) [27].

Continuous flow nitration of a few important arenes using the standard nitrating mixture in a CYTOS microreactor was carried out by Panke et al. [27]. The nitration product of 1-methyl-3-propyl-1*H*-pyrazole-5-carboxylic acid is an intermediate for the “life-style drug” Sildenafil^®^ (Scheme 1). Similarly, nitration of 2-methylindole, pyridine *N*-oxide and toluene (also with an acetyl nitrate Ac_2_O/HNO_3_ mixture) were conducted. The conventional procedure for the conversion of 2-methylindole (**4**) into 2-methyl-5-nitroindole (**5**) relies on the addition of NaNO_3_ in H_2_SO_4_ to the starting material (Scheme 2) over a period of 1.5 hours. This helps to maintain the internal temperature at 0 °C and gives 80% yield. The continuous laboratory scale process required only 0.8 minutes at 3 °C to obtain 70% yield of the desired nitro derivative. For the nitration of pyridine *N*-oxide (**6**), which requires a higher temperature (~120 °C), the approach using a microreactor resulted in a yield of 78%, an improvement compared to the conventional approach (72% yield, Scheme 3). The authors carried out the continuous nitration of toluene (**8**) with the nitrating mixture H_2_SO_4_/HNO_3_ and with acetyl nitrate generated in situ from HNO_3_ and Ac_2_O (Scheme 4). The first method resulted in >98% conversion and 48%, 36% and 8.2% yields, respectively, for *ortho*-, *meta*- and *para*-mono-nitro isomers. The second method led to a complete conversion with 54%, 39% and 2.7% yields, respectively. The yield of the secondary nitration products was smaller in the presence of acetic anhydride. This observation is important as nitrations that involve acetic anhydride are inherently unsafe, yet the range of conversion and the yields that are achieved will be different depending upon the mechanism.

**Figure 3:**
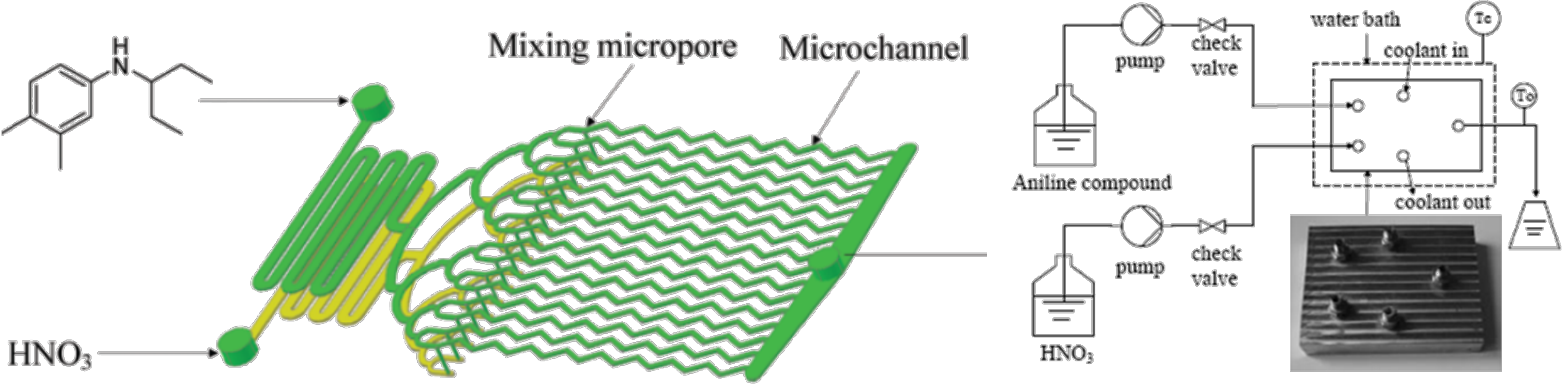
Graphical presentation of a microreactor used for double nitration and the schematic of the experimental setup. The microreactor includes a built-in facility for heat transfer. Reproduced with permission from [41]. Copyright 2013 The Royal Society of Chemistry.

In general, the dinitroaniline derivatives are produced by nitration of anilines. Chen et al. [41] studied the one-step dinitration that yields *N*-(1-ethylpropyl)-3,4-dimethyl-2,6-dinitroaniline. Under conventional conditions of nitration, oxidation or over-nitration of aniline derivatives is unavoidable, as the reaction rates are strongly limited by interfacial mass transfer due to biphasic conditions. The selectivity of the isomer of interest can be increased by avoiding any pre-protection of the amino groups of aniline derivatives. All the experiments have been carried out in a microreactor (0.2 mL volume) with very high heat and mass transfer coefficient allowing excellent temperature control (<±2 °C) (Figure 3). In the conventional two-step approach the aniline solution (30 wt %) is treated with diluted nitric acid as the first step [44], and after isolation the intermediate is again treated with additional concentrated nitric acid as the second step. The reaction time is 4 hours and the reaction gives 89% yield of pendimethalin (*N*-(1-ethylpropyl)-3,4-dimethyl-2,6-dinitroaniline) and *N*-nitrosopendimethalin with a molar ratio of 7:3. Higher concentrations of nitric acid gave higher degrees of conversion. When carried out in a microreactor the same reaction gives 100% conversion and 97% yield with 3 mol equivalents of nitric acid at 60 °C. The process could be scaled-up up to 432 tons per year and the protocol has been adapted for other aniline derivatives.

**Scheme 5:**
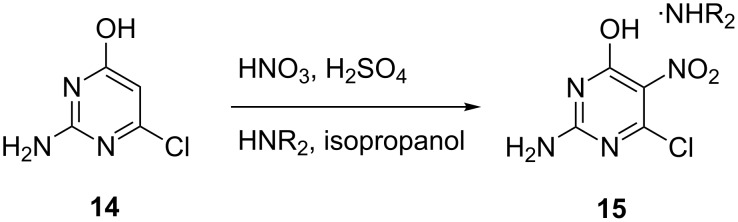
Nitration of 2-amino-6-chloro-4-pyrimidinol (**14**) [25].

Continuous flow nitration of 2-amino-6-chloro-4-pyrimidinol (**14**) for the synthesis of 2-amino-6-chloro-5-nitro-4-pyrimidinol (**15**) and its stable diisopropylamine salt using 90% nitric acid in sulfuric acid are reported by De Jong et al. [25] (Scheme 5). The substrate is dissolved in sulfuric acid and the reaction is carried out in a translucent Teflon tube immersed in a constant-temperature bath. Typical residence time of the reaction mixture in the tube was approximately 2.5 min and the outlet mixture was quenched in a tank containing cold water.

**Scheme 6:**
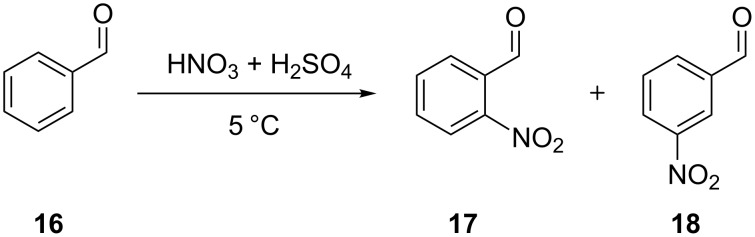
Nitration of benzaldehyde (**16**) [35].

**Scheme 7:**
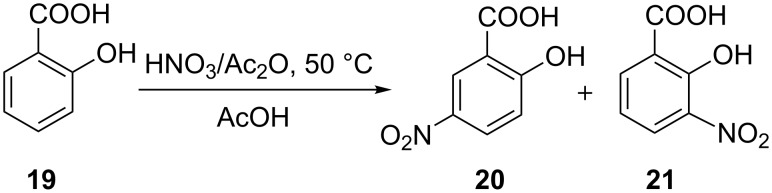
Nitration of salicylic acid (**19**) [30].

In another study, Kulkarni et al. [35] showed that the nitration of benzaldehyde (**16**) can be carried out in a safe manner in a continuous mode using a microreactor system (Scheme 6). The performance of this two phase reaction critically depended on the choice of a micromixer. The availability of high heat transfer area helped to increase the reaction temperature to reduce the reaction time to 2 min. Efficient mixing, which can be achieved by using structured micromixers, i.e., caterpillar micromixer, was favored over the simple T-micromixer. The experimental setup consisted of two syringe pumps, which pump the reactants – a nitrating mixture and benzaldehyde – through 20 mL glass syringes connected to SS316 tubes by a glass-to-metal PTFE connector. After the micromixer a hastelloy tube (1.38 mm i.d. and 6 m long) acted as a residence time unit immersed in a thermostat maintained at 5 °C. The contact angles of the aqueous phase on the SS316 ensured that it remained in the continuous phase, while benzaldehyde was present in the form of discontinuous slugs. The authors observed that the rate constant of the reaction leading to the formation of the *meta*-isomer was higher and the rate of change in the ratio of isomers increased with increasing amounts of nitric acid. The greatest mol fraction of the *meta-*isomer **17** was obtained when the HNO_3_ was employed in 3.5 equivalents of **16**. The same reaction was conducted with a nitrate mixture composed of sulfuric acid, nitric acid and acetic anhydride, which led to a homogeneous system. However, an over-oxidation of **16** was encountered due to the presence of acetic anhydride, so that benzoic acid was formed. The poor solubility of benzoic acid in the aqueous phase led to an immediate clogging of the tubular reactor. This undesired clogging occurred under a wide range of temperature and residence time conditions thereby preventing a successful nitration. This example illustrates that a homogeneous reaction mixture is not a sufficient condition to successfully transform a known nitration into continuous mode, the handling of the solubility of the undesired solid has to be considered as well. This issue was finally dealt with by dissolving the benzoic acid in a suitable solvent (*n*-hexane), which also reduced the reaction time to a few seconds. However, even though the conversion of benzaldehyde was complete, the presence of acetic anhydride resulted in a significant change in the isomeric ratio and yielded many undesired products. In another example, continuous flow nitration of salicylic acid (**19**) with HNO_3_/AcOH was performed by Kulkarni et al. [30] in a SS316 tubular microreactor. Compound **19** was completely converted to mono-nitro derivatives in less than 7 min, and afforded 5-nitrosalicylic acid (**20**) as the major product (Scheme 7). A large excess of acetic acid in the reaction mixture was necessary to avoid precipitation of the desired product **20**. The authors reported the formation of byproducts when the reaction was conducted at higher temperatures. Continuous operation for 2 hours consistently yielded the same composition at the outlet. In a more detailed work, these authors showed the importance of heat transfer for the continuous flow nitration of salicylic acid, as poor heat transfer can give a higher conversion rate but lower selectivity for the desired product. Contrary to a patent description [45], glass is less suited than metal SS316 or Hastelloy due to lower thermal conductivity. Thus, the choice of material of the reactor plays a significant role in controlling the yield and selectivity of the desired product.

**Scheme 8:**
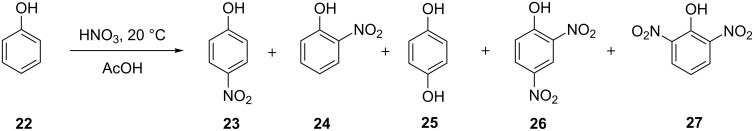
Nitration of phenol (**22**) yielding mono-nitro isomers **23** and **24** as main products, hydroquinone (**25**), dinitrophenols (**26** and **27**), and polymeric side products.

Ducry and Roberge [29] used a glass microreactor with <0.5 mm channel width and 2.0 mL internal volume for the continuous flow nitration of phenol (Scheme 8). The nitration was investigated with a wide range of phenol concentrations as well as different equivalents of HNO_3_. Acetic acid was used as a solvent. Experiments were also carried out with 10% water instead of an organic solvent. In the absence of a solvent the results from the continuous flow experiment turned out to be superior to batch experiments with a significant increase in the formation of mono-nitro products. The authors reported that with 1.4 equivalents of nitric acid at 20 °C a yield of 77% of mono nitrophenols can be achieved.

**Scheme 9:**
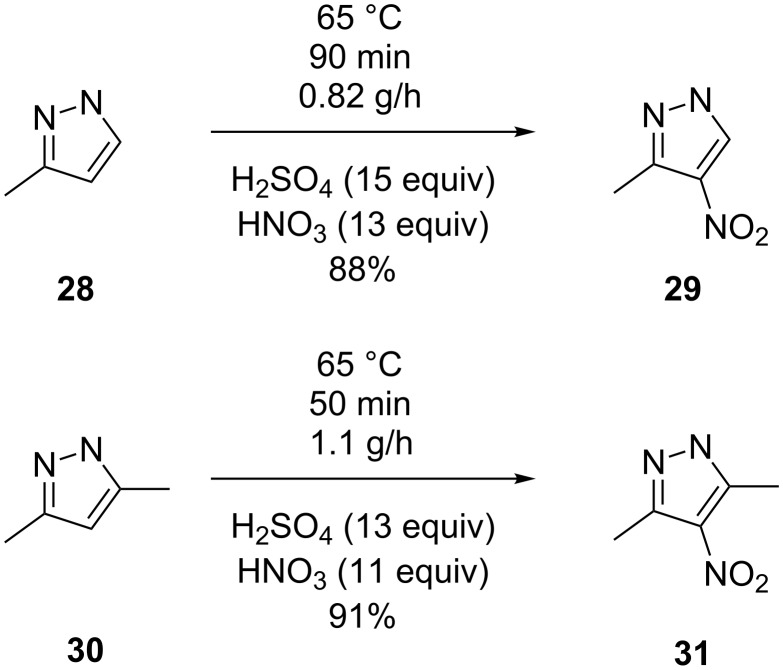
Synthesis of 3-methyl-4-nitropyrazole (**29**) and 3,5-dimethyl-4-nitropyrazole (**31**) [31].

**Figure 4:**
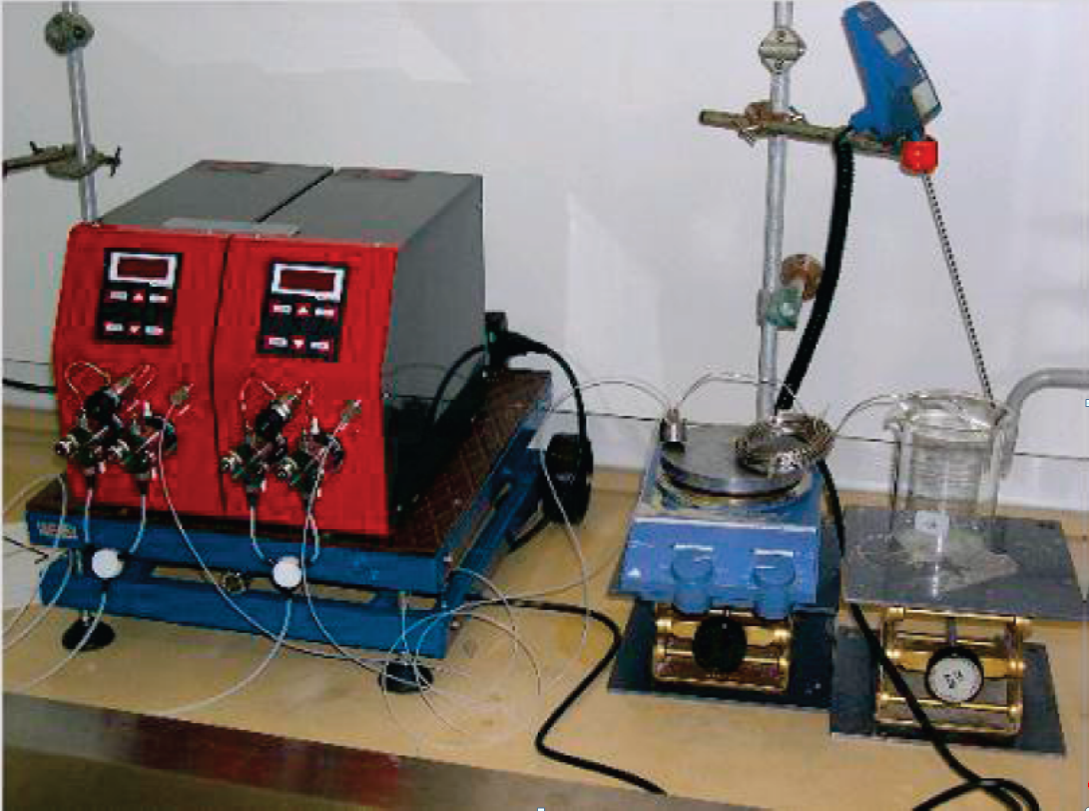
Photograph of the experimental setup for the synthesis of alkyl-nitropyrazoles. IMM’s SIMM-V2 micromixer was used to ensure better mixing at the cost of pressure drop. Reproduced with permission from [31]. Copyright 2009 The American Chemical Society.

An important example of continuous flow nitration leading to alkyl nitropyrazoles was reported by Pelleter and Renaud [31]. The nitration products 3-methyl-4-nitropyrazole (**29**), 3,5-dimethyl-4-nitropyrazole (**31**) and 3-ethyl-4-nitropyrazole (**32**) were obtained with nitrating mixture and were expected to show detonating properties under severe confinement (Scheme 9). This flow synthesis did not allow the pressure inside the reactor to undergo rapid variations in a short time, thereby increasing the safety of this synthesis approach. The experimental setup for this nitration is shown in Figure 4. The set-up is similar to the systems used in the literature except that a back pressure regulator was used to ensure that any pressure variations during synthesis are suppressed to avoid any critical situation. It is important to note that the pyrazole was nitrated after dissolving it in the sulfuric acid. For a reactor of 10 mL volume, the temperature was maintained at 65 °C, and the hydrolysis was carried out by dropping the reaction mixture into a cold aqueous solution saturated with potassium carbonate. The residence time for the synthesis of compounds **29** and **31** was 90 minutes, while it was 25 minutes for compound **32**. The formation of dinitro derivatives can be prevented by a strict temperature control. While the authors indicate that a higher amount of products may be synthesized by increasing the number of micromixers and extending operating hours, specific calculations on the economic viability are not given. A longer residence time leads to lower flow rates and thus to laminar flow conditions. In spite of a higher heat transfer area, such conditions do not offer high heat transfer rates and result in axial dispersion. Consequently, restricting the temperature below 65 °C can avoid the dinitro products, ensuring that the reaction mixture is locally homogeneous. One of the ways to reduce the axial dispersion is a segmented flow, which is implemented by using an inert gas or immiscible inert liquid. However, under such conditions it is essential to ensure a continuous mixing along the length of reactor. In this report, the authors used excess acid. While it may be required for a reaction to keep the entire mixture homogeneous, the overall process suffers from a neutralization step to isolate the product. However, since the large-scale synthesis of nitropyrazoles is not a safe reaction under batch conditions, the continuous flow synthesis is the process of choice.

Henke and Winterbauer [46] reported a corrosion resistant microreactor built up of PTFE and tantalum for the simulation of different nitration reactions. The microreactor proposed for this adiabatic nitration included components made in tantalum and PTFE connected in series. In the reactor, the dimensions of the components vary along the length and thus they induce mixing, initially of sulfuric acid with nitric acid and later with benzene. Orifices with different diameters induce different flow regimes in the reactor. The authors claim this microreactor has infinite scalability, as it is based on the addition of different components in sequence. In one of the first reports on the continuous flow nitration of benzene without the use of sulfuric acid Othmer et al. [47] used a column (1 inch diameter and 1.22 m long) that was attached to a reflux condenser, which led into a decanter. It was reported that the continuous nitration plant is advantageous over the batch process in terms of greater production capacity per man-hour and per square foot of floor space. Furthermore, the mixed acid can be avoided. The raw material costs are also lower due to a virtually quantitative conversion. However, the major disadvantages of the continuous process are associated with the comparatively higher costs of the reactor and additional equipment materials, which are required to be composed of stainless steel or other resistant material.

Dummann et al. [43] used a capillary-microreactor for studying the nitration of a single aromatic ring. The authors did not provide details about the specific aromatic substrate. Nevertheless, the observations are useful in giving a fair idea on what to expect and what to consider from a nitration reaction in general. In their approach they restricted their analysis to the nitration of monocyclic aromatic substrates with mono and dinitro derivatives as products and a phenolic byproduct. The experimental setup consisted of a Y-piece followed by a capillary-microreactor (PTFE capillary ~ i.d. = 0.5 to 1.0 mm) immersed in a thermostated jacket. This jacket was maintained at a constant temperature between 60 to 120 °C by using a countercurrent flow of silicone oil at high flow rates. This setup is similar to the one shown in Figure 2, but adds a quenching line to quench the reaction inline by rapidly reducing the temperature to 20 °C for a specific section of the reaction tube. The reaction is carried out under pressure (~4 bar) to avoid any degassing from the reaction mixture. From various experiments under different conditions the authors observed that increasing the flow velocity actually increases the degree of conversion along with an increase of the byproduct due to parallel reaction and reduces the byproduct due to sequential reaction. Consequently, controlling the residence time distribution and the heat transfer rates allows for a restricted formation of byproducts from the sequential reaction. With an increase of the fluid velocity the overall mass transfer coefficient was found to increase. It seems that a higher fluid velocity facilitates the rapid mixing of the two reagents and thereby enhances the heat generation rate in the system. Given a constant heat transfer area, although the heat transfer coefficient increases with an increasing velocity, a higher rate of heat generation causes a rise in the local temperature thereby enhancing the rates for parallel reactions. A control of the residence time supports the avoidance of byproducts from sequential reactions. This observation is common for all systems and thus it is necessary to optimize the energy balance in the system depending on the heat transfer rates, the heat of the reaction and the inlet flow rates.

**Scheme 10:**
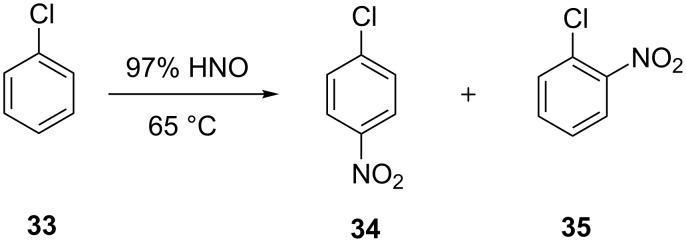
Nitration of chlorobenzene (**33**) [23].

**Figure 5:**
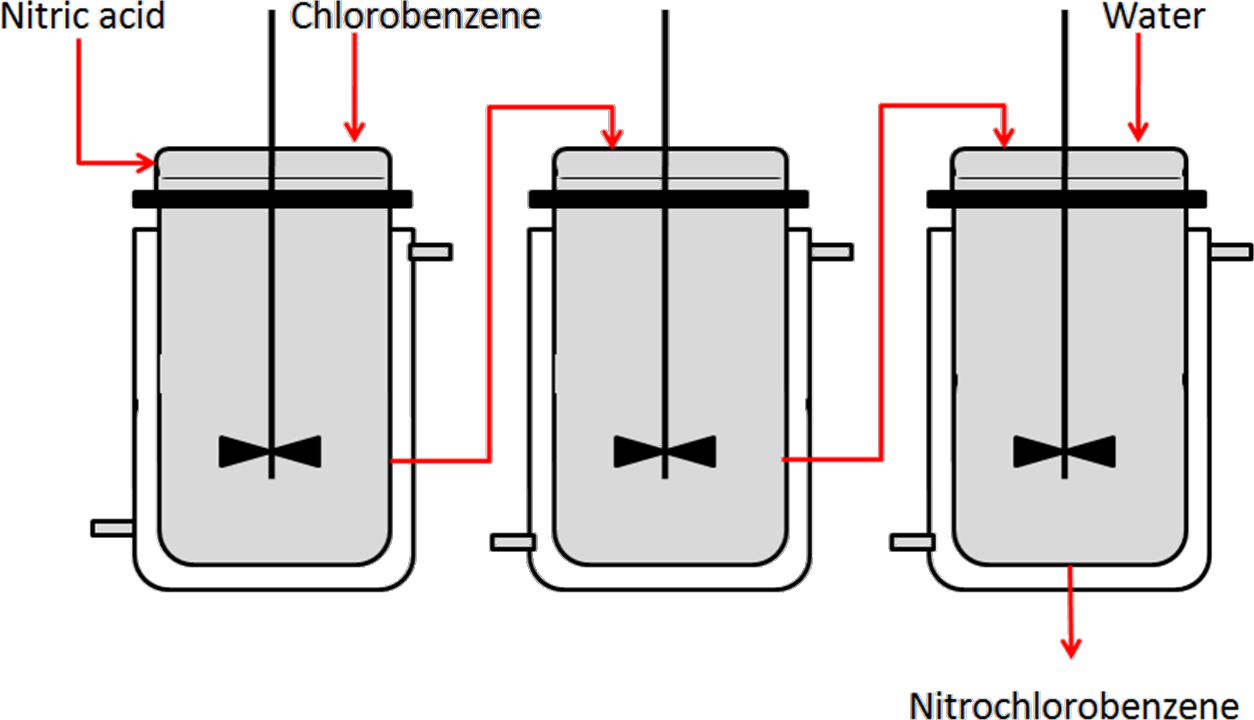
Continuous flow nitration of chlorobenzene (**33**) with nitric acid in a sequence of continuously stirred reactors [23].

Veretennikov et al. [23] reported on the continuous flow nitration of chlorobenzene (**33**) for the production of mono-nitrochlorobenzene (**34**, **35**) by using 75–97% nitric acid in a series of continuous stirred reactors (Scheme 10, Figure 5). The reactors were made from 1Cr18Ni10Ti steel and the reactor volume was 60 mL. The experiments were carried out over a range of 65–85 °C at a molar ratio of nitric acid to chlorobenzene in the range of 1.5 to 3. The authors have reported the highest yield of mono-nitrochlorobenzene (98.2%) at a molar ratio of 3 with 90% nitric acid, 75 °C and 45 minutes residence time. The third reactor is used for hydrolysis by water addition. The authors have quantitatively measured the role of hydrolysis on the basis of the precipitated products and suggested a method for the selective separation of nitrobenzene **34** by exploiting the principle of preferred solubility domain.

In a similar approach, a single continuous glass/Teflon stirred tank (CSTR) with a volume of 1.06 L (*d* = 9.5 cm, *h* = 15.1 cm) was used by Quadros et al. [48] for the adiabatic nitration of benzene witth mixed acids. The pilot-scale continuous stirred reactor was operated at a temperature range of 80–135 °C with 4.9–5.6 wt % HNO_3_. At fixed residence time and fixed concentration of sulfuric acid, adiabatic rise in the local temperature was observed. The fraction of mono-nitrobenzene in the reaction mixture increased with increasing impeller speed, which clearly indicated that the reaction was mass transfer limited. The authors showed that the kinetic parameters are a function of the sulfuric acid concentration, which acts as a catalyst. While the adiabatic operation was shown to work in this case, the set of optimal conditions or the effect of various parameters on the yield of the product was not reported.

**3.1.2 Nitration with fuming nitric acid.** The use of sulfuric acid or any other acid in combination with nitric acid leads to the rapid generation of nitronium ions in their ionic form stabilized by water. However, the application of these nitration conditions under continuous flow conditions in a pilot plant setup or commercial large-scale process, results in economic as well as environmental problems as the sulfuric acid has to be neutralized and substantial amounts have to be separated, which entails additional process equipment of the plant. Fuming nitric acid allows for the avoidance of sulfuric acid and also reduces the chemical footprint of the nitration systems. We discuss here the flow nitration with fuming nitric acid without reviewing the mechanism in detail.

**Scheme 11:**
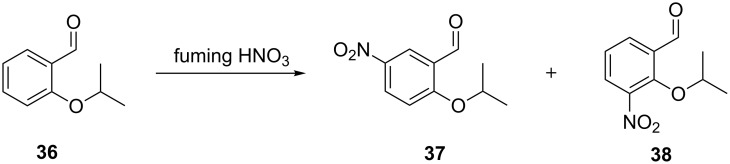
Nitration of 2-isopropoxybenzaldehyde (**36**) by using red fuming nitric acid [37].

**Figure 6:**
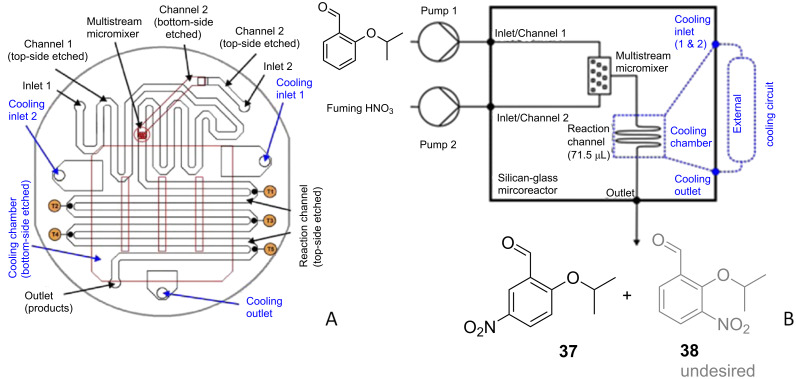
Silicon-glass microreactor by Knapkiewicz et al. [37]. (A) Layout of the microreactor with a built-in micromixer, (B) schematic of the experimental setup. Reproduced with permission from [37] Copyright 2012 The American Chemical Society.

The continuous flow nitration of 2-isopropoxybenzaldehyde (**36**) with red fuming HNO_3_ has been reported for the first time by Knapkiewicz et al. [37] (Scheme 11). The product 2-isopropoxy-5-nitrobenzaldehyde (**37**) is an intermediate to obtain a nitro-substituted Hoveyda–Grubbs catalyst. Scale-up based on the conventional batch approach yielded a higher extent of the undesired regioisomer **38** (37% rise than the laboratory scale batch). The selectivity of the desired product **37** was improved under flow conditions. A continuous flow silicon-glass microreactor equipped with multiple functionalities such as multistream micromixer, reaction channel, large-area cooling chamber, and five integrated miniature temperature sensors was used (Figure 6). At the reactor outlet compound **37** was collected in ice water for 120 min followed by separation and isolation of the organic layer. While the batch experiment yielded only 30% of the desired nitro derivative **37**, the continuous flow process yielded close to 67% yield at a comparable scale.

**Scheme 12:**
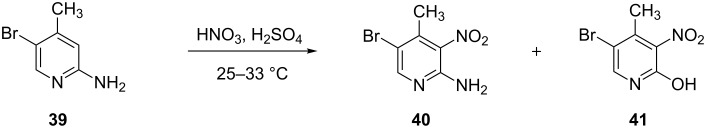
Synthesis of nitropyridine (**40**) [39].

Gage et al. [39] developed a simple and practical flow reactor to produce nitropyridine in an exothermic nitration reaction (Δ*H* ~ −167 kJ/mol). Their approach is suitable for large-scale productions of up to hundreds or thousands of kilograms of nitroaromatics, Typically nitropyridine is prepared in batch mode by dissolving 5-bromo-2-amino-4-methylpyridine (**39**) in concentrated sulfuric acid, to which fuming nitric acid is added at 25−33 °C. The reported yield of the desired product is 52−55% (Scheme 12).

**Figure 7:**
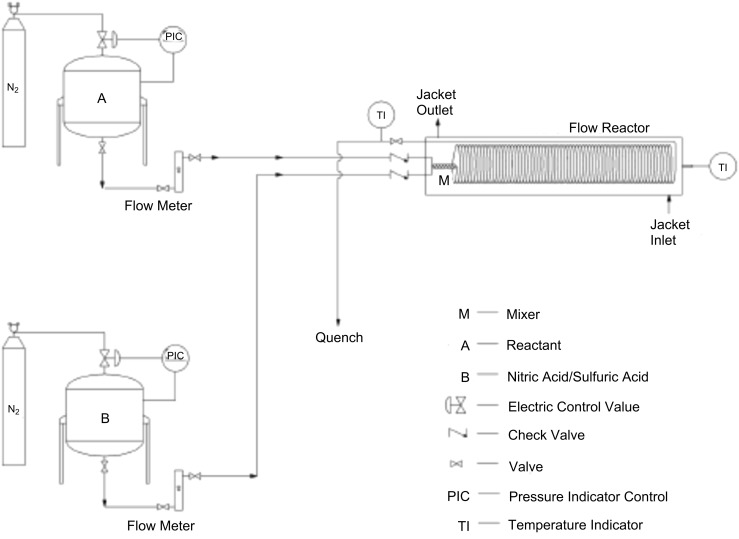
Schematic of the experimental setup involving a pressure based charging system [39]. Reproduced with permission from [39]. Copyright 2012 The American Chemical Society.

The experimental setup included the feed vessels, the mixer, the residence time loop, and a collecting vessel, all of which are connected by stainless steel tubing and ports (Figure 7). The reactant **39** was dissolved in H_2_SO_4_ (1/3 wt/wt) and HNO_3_/H_2_SO_4_ (~1/12 wt/wt). 97% conversion is achieved in 20 minutes residence time at 50–55 °C reactor temperature.

Scale-up was possible by using a larger apparatus consisting of a 45 m long reactor tube (15 mm i.d.) with a total volume of 7.95 L. The flow of reactants was achieved by applying nitrogen pressure on the feed tanks (Figure 7). The authors demonstrated their concept for larger flow rates (70 mL/min) with an isolated yield of 59% for nitropyridine **40**. However, the approach requires a pre-calibration of the flow meters depending upon the density of the fluids. Although the reported variation in the flow rates was 4%, this results in a variation of the local concentration, so that an inhomogeneous conversion pattern is obtained. Obviously, the scale-up experiments gave much lower yields than the theoretical value, which indicates that temperature control is an important issue to ensure the desired heat transfer. Furthermore, uniform distribution of residence time and continuous product monitoring is necessary to avoid a runaway reaction in the flow reactor.

**Scheme 13:**
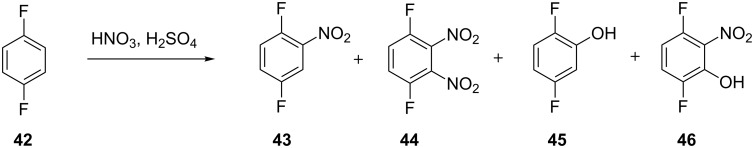
Nitration of *p*-difluorobenzene (**42**) [40].

Yu et al. [40] reported on the continuous flow process for the synthesis of 2,5-difluoronitrobenzene (**43**) via nitration of *p*-difluorobenzene (**42**) with a nitrating mixture composed of 2.0 equiv concentrated sulfuric acid and 1.1 equiv fuming nitric acid (Scheme 13). Two different approaches were tested. In the first approach, the reactants were continuously mixed by using a T-mixer followed by a tubular reactor maintained at a constant temperature (10–15 °C). Hydrolysis was carried out with ice water (~20 mol per mol of the reactant). The usage of a single tube facilitated a higher isolated yield of isomers **43** and **44** with increasing residence times. However, the conversion was only 76% and an increase of the temperature entails a higher amount of byproducts. Although the authors regard the phase separation accountable, usually it remains in the slug flow unless the aromatic substrate is soluble in the nitrating agent. Typically the slug flow gives excellent interfacial mass transfer rates due to continuous surface renewal as the slugs travel through the tubular reactor. Thus, the phase separation actually may not be the reason for the lower conversion. An increased conversion could be possible by further increasing the residence time or by providing the necessary concentration of nitric acid to achieve the desired D.V.S. value for this system. The mass transfer rates can be enhanced by increasing the flow rates. However, for a given tubular reactor at a constant flow rate the reaction rates can be enhanced by increasing the temperature. Furthermore, a smaller concentration of nitronium ions also affects the reaction rates, provided the nitric acid is used in significant excess [16]. The residence time was varied by using tubes of different lengths at identical flow rates. This approach is particularly important because a change of flow rates to vary the residence time usually entails different implications, such as influencing the mixing and heat transfer during mixing of the reagents. At a fixed tube length smaller residence times are achieved by increasing the flow rates leading to a better mixing and a better convective heat transfer. However, high flow rates lead to an increased overall heat generation rate for a fixed heat transfer area, which can enhance the reaction rates. On the other hand, a variation of the tube lengths for fixed flow rates gives more consistent data as the inlet conditions are fixed and the flow pattern or the velocity profiles in the reactor remains unchanged due to constant flow rates.

**Figure 8:**
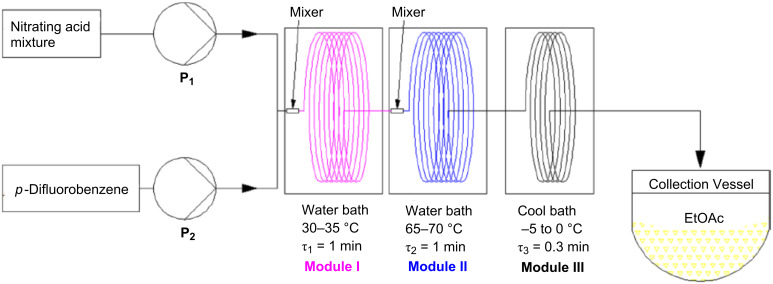
Schematic of the flow reactor arrangement. Reproduced with permission from [40]. Copyright 2013 The American Chemical Society.

In order to achieve complete conversion the experimental setup (Figure 8) involved three sections. The first two sections were run at a residence time of 1 min while the third one was run with a residence time of 20 s. Each section was maintained at a different temperature, more specifically 30–35 °C, 65–70 °C and −5 to 0 °C, respectively. The role of the last segment was to quench the reaction. This setup is reported to achieve 98% yield for the desired product at a rate of 6.25 kg/h. Lower reaction rates due to the continuously reducing concentration of nitric acid along with the reactor length was compensated by an increase of the temperature and also by keeping an optimal residence time. The observations indicated that the smaller diameter reactor tubes yielded more side products. On the other hand, a larger diameter reactor led to lower conversion rates. Although the authors have recycled and reused the nitrating mixture by adding make-up nitric acid for some of the experiments, further optimizations are necessary to achieve an economical process.

In a novel approach, Antes et al. [28] disclosed (i) online monitoring of nitrations in microreactors by using FTIR microscopy as well as thermographic methods and (ii) the continuous separation and detection of the nitration products by using HPLC. In order to avoid a post treatment of the mixed acids in the conventional approach, the authors used fuming nitric acid. The experimental setup comprised three independent pumps for toluene, fuming nitric acid, and ice-water. The reaction took place in a silicon microreactor consisting of nine reaction channels in parallel (0.25 mm channel width) with a G-shaped micromixer. The design has G-shapes in alternate directions, which cause a continuous change in the flow direction, splitting and recombining. The highest yields for mono-nitrotoluenes (89–92%) were obtained at −10 °C and in a residence time of 3 s by using 2.56 equivalents of nitric acid per mol of toluene. The experiments using the microreactor yielded a 10% rise in the *para-*isomer compared with the industrial batch process. A similar approach was used for the nitration of thioureas, and the authors showed by online FTIR spectroscopy that the mechanism of the nitration of thioureas is based on subsequent nitrosation and nitration steps.

**Scheme 14:**
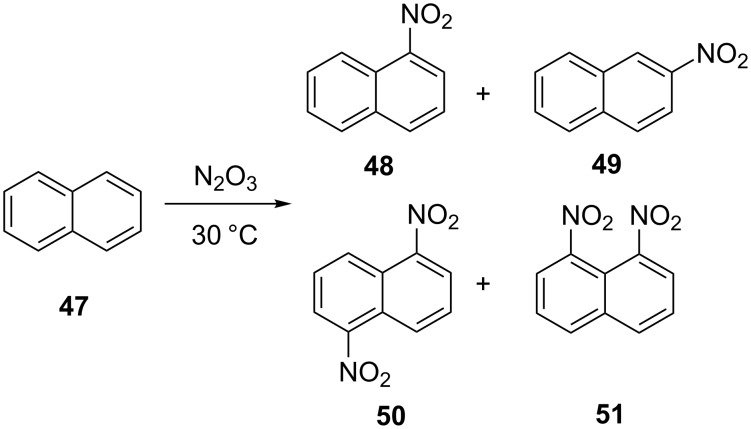
Nitration of naphthalene (**47**) [34].

The same group also analyzed continuous processes to perform the strongly exothermic nitration of naphthalene (**47**) (Scheme 14) with N_2_O_5_, both in the gas phase and in the liquid phase [49–50]. The authors reported that the nitration with the conventional batch method requires a cooling to temperatures between −50 to −20 °C, while the same reactions can be carried out in a microstructured flow reactor at 30 °C with a residence time of just 3 s. The outlet product composition contains both, the mononitro derivatives as well as the dinitro compounds. In macroscopic batch reactors the isomer ratio of **50** and **51** is always ca. 1:3.6, while the flow synthesis yielded more 1,5-dinitro compound **50** (**50**:**51** ~1:2.8). The isomer ratio of mono-nitronaphthalene products **48**:**49** could also be changed to 32:1 in a microreactor, while the typical isomer ratio in industrial processes is 20:1.

**Figure 9:**
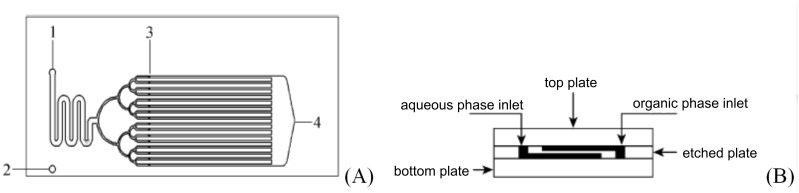
Structure of the microreactor. (A) Top view (1, 2 – inlets, 3 – mixing points, 4 – outlet). (B) Lateral view of the inlets for the microreactor. Reproduced with permission from [34]. Copyright 2009 Elsevier.

In another example of producing energetic materials using microreactors, Shen et al. [34] reported the two phase nitration of isooctanol and a mixed acid to produce 2-ethylhexyl nitrate. A SS316 microreactor was used, in which the mixing of the reagents occurred after the distribution of the first reactant (Figure 9), and the reaction took place in 78 mm long parallel microchannels (0.5 mm × 0.5 mm) connected to a common outlet. The hydrolysis and instantaneous termination of the nitration reaction occurred outside the microreactor by rapid dilution of the reaction mixture with an ice-water mixture at 0 °C. The experimental observations showed that for an identical residence time and in the presence of 2% H_2_O (by mass), the conversion of isooctanol was unaffected by changing the sulfuric acid concentration from 67% to 86% (by mass). The amount of sulfuric acid and the residence time corroborated the expected trends in this two phase nitration reaction. However, the authors have justified their observations on the basis of the interfacial mass transfer rates. For this system, the range of flow rates covers different flow regimes, namely, the parallel flow with a smooth interface, the parallel flow with a wavy interface, and the chaotic thin striations flow. Since the overall reaction rates are controlled by interfacial mass transfer, an increase in the linear velocity results in higher average reaction rates. An excellent mixing and control on mass transfer rates allows carrying out this reaction safely and stably in the specially designed microreactor at 25-40 °C with 98.2% conversion of isooctanol an no byproducts.

**Scheme 15:**
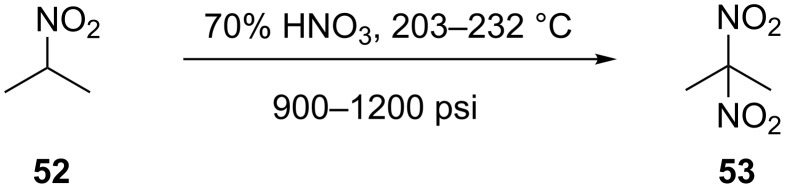
Nitration of 2-nitropropane (**52**) [38].

**3.1.3 Vapour phase nitration.** In an early study on the continuous flow nitration of 2-nitropropane (**52**), Denton et al. [22] demonstrated that high pressure and high temperature conditions, i.e., 900 to 1200 psi and 203–232 °C, using an equimolar nitric acid (70%) gives about 50% yields per pass (Scheme 15). Their reaction assembly consists of a stainless steel preheater tube (outer diameter of 6.24 mm) passed through a 40 inch long 20 mm outer diameter. The reactor was packed with glass beads to increase the contact surface and mixing [51]. A water condenser was used for quenching the reaction by cooling, followed by a pressure reduction to atmospheric pressure by using a needle valve, which was further cooled by means of an ice condenser followed by a gas–liquid separator.

Similar to the aforementioned study, Löwe et al. [38] reported the vapor phase nitration of propane in a multistep microreactor in a highly sophisticated and safe system. Different steps in the process are integrated in a single reactor. While the reaction occurs at high temperature (380–450 °C), the quenching was performed by hydrolysis as well as by rapid cooling of the diluted reaction mixture. The term per-pass conversion is used when the unreacted reactant is recycled back to the inlet. If no such recycling is carried out, the per-pass conversion is estimated on the basis of the total reactant used in the reaction. The per-pass conversion was maintained at 2% at the cost of selectivity for 2-nitropropane (**52**), which gets significantly affected by the residence time. The authors employed an integrated single flow reactor composed of a special stainless steel alloy (1.4361, X1CrNiSi18154). Electrical heaters and two integrated water coolers were used for heat transfer in different sections of the integrated reactor. The integration of these functions in one setup facilitates the handling of hazardous chemicals at higher temperatures and avoids any release of toxic gases into the environment.

A corrosion resistant glass syringe pump was used for pumping the nitric acid to the evaporator section, which upon mixing with pre-heated propane proceeded to the heated reaction section, whose temperature was maintained between 380 and 455 °C. At the outlet of the reaction section, the reaction mixture was diluted with water by an integrated caterpillar micro mixer and subsequently sparged through an ice-water filled flask to condense volatile nitric compounds outside the integrated microreactor. Reaction optimization was conducted over a wider range of mol ratio of the reactants (propane:HNO_3_ ~ 0.5 to 6), different residence times (0.4 to 2.5 s), and gas flow velocities between 5 and 0.9 m/s. An analysis of the exit stream revealed the formation of 2-nitropropane, 1-nitropropane and nitroethane. At a residence time of 1 s, the yield of 2-nitropropane was independent of the temperature. However, an increase in temperature entailed a decrease in the yield of 1-nitropropane and vice versa for nitroethane. At a constant temperature the mol fraction of 2-nitropropane was (i) independent of the residence time at a constant inlet mol ratio and (ii) decreased with an increasing inlet mol ratio of propane to nitric acid. The authors concluded that the microstructured reactors are not advantageous for this case, because the rapid quenching of the radical chain mechanism causes a lower conversion. The important consequences of this work are (i) the development of an integrated microreactor concept, (ii) issues related to safety and operation are improved significantly to ensure that the toxic chemicals do not escape throughout the process, (ii) the authors conclude by saying that a holistic process design approach should not be overlooked without focusing entirely on using the “micro reactor” concept.

**3.1.4 Nitration with solid acid catalysts.** The continuous flow vapour phase nitration using a solid acid catalyst has also been explored [26]. It is known that the solid acid catalysts, i.e., ZSM and other zeolite catalysts, can improve the selectivity of *p*-nitrotoluene in a conventional reactor using a mixed acid as the nitrating agent [52–53]. Continuous flow nitration of toluene in a packed bed microreactor using concentrated nitric acid as the nitrating agent was reported by Halder et al. [54]. Different ‘solid acid’ catalysts were studied to identify the right catalyst that would yield better isomer distribution. In such cases, self-protonation of nitric acid drives the reaction rapidly, and the isomer ratio of nitrotoluenes remains similar to the standard nitrating mixture and without byproducts. However, in the microreactor, concentrated nitric acid reacted very rapidly in the absence of any sulfuric acid or a solid acid catalyst. Nitric acid and toluene were brought into contact by using a SS316L T-mixer (1.58 mm inner diameter) at room temperature. The immiscible reactants were passed through a tubular microreactor (SS316L, i.d. = 0.775 mm, *l* = 8.5 cm) packed with different solid acid catalysts (Stevens catalysts A and B, ZSM-5-280). In the fixed bed microreactor, 13 mg of catalyst (*d*_p_ = 75–150 μm) was packed over 6.0 cm distance, after which the remaining reactor length was filled with smaller inert glass beads (20 mg, *d*_p_ = 63–75 μm) to prevent the carryover of any fine catalyst particles and subsequent clogging of the filter placed at the end of the reactor. The nitration was terminated by collecting the product in a sodium carbonate solution. Most of the conversion took place outside the solid acid catalyst bed. Moreover, the nitration of toluene with 90 wt % nitric acid using a microreactor was found almost entirely preceded under kinetic control. However, with only nitric acid as the nitrating agent, the nitrotoluenes were generated in a low yield. The formation of water adversely affected the availability of nitronium ions. This can be overcome by using sulfuric acid which reacts with the formed water. Thus, the optimal conditions can be achieved by increasing the temperature to a limit that does not lead to runaway conditions.

Recently, Yang et al. [32] studied the nitration of benzene in a continuous flow microreactor loaded with a microfiber structured Nafion/SiO_2_ solid acid catalyst. The catalyst was prepared by using the solgel technology that leads to coating on the surface of the microchannel reactor (thickness ~200–400 nm particles). Consequently, a slightly porous surface is formed that is able to improve the mass transfer rates in close proximity to the reactor wall. At 75 °C and a 36% (w/w) loading of the microstructured solid acid catalyst, the authors have reported 44.7% conversion of benzene with a 99.9% selectivity of nitrobenzene. At an equivalent conversion level, the microstructured Nafion/SiO_2_ catalyst was 600 times more effective in terms of activity per acid site compared to the liquid sulfuric acid. However, in order to practice this approach, the following information may be useful: (i) the longevity of the catalyst (turn over number) under different situations, (ii) the stability of the catalyst and the support under different aqueous and organic compositions, and (iii) the method of catalyst deposition.

#### 3.2 Analysis of recent patents

**Figure 10:**
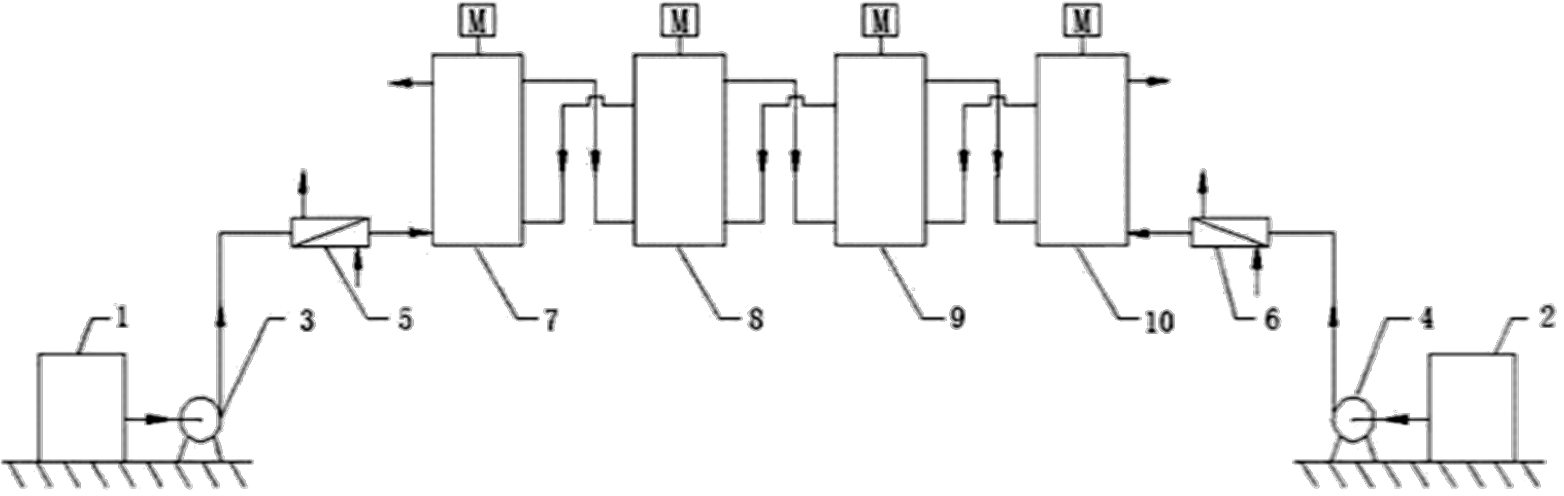
Schematic of the continuous nitration system reported in CN103044261A [56].

The continuous flow nitration of naphthalene-2,7-disulfonic acid leading to 1,8-dinitronaphthalene-3,6-disulfonic acid has been disclosed in CN102320995A [55] using mixed acids (Figure 10). The inventors have studied the performance by varying the reaction temperature between 35 and 120 °C and the reaction time up to 120 minutes. Examples were reported for the nitration at 50 °C for a residence time of 60 minutes that allowed an adiabatic temperature of less than 160 °C. Additional examples with a tubular reactor equipped with packing Raschig rings and SV static mixers are also mentioned.

In a very interesting method disclosed in CN103044261A [56], continuous centrifugal extractors were used for nitrations. The simultaneous addition of the mixed acid solution as a heavy-phase and the raw material (substrate) as a light-phase to different centrifugal extraction separation devices facilitates the reaction and extraction to be achieved simultaneously. The system works in a counter current mode. The reaction temperature and the rotating speed of the centrifuge were varied between 10 to 160 °C and 800–2000 r/min, respectively. The invention claims to achieve a continuous compact automated device that can be used for the large-scale production and isolation of the organic and aqueous streams. The inventors reported the nitration of aromatic hydrocarbons, namely benzene, toluene or chlorobenzene and polyhydric alcohols such as glycerol or 1,2,4-butanetriol. The flow reactor size is claimed to be reduced to 0.6 to 1% of the conventional batch reactor for identical production capacity.

In a novel approach, US20130197268A1 [57] discloses an invention for the continuous nitration of benzene with a mixture of nitric acid and sulfuric acid under adiabatic conditions for producing nitrobenzene. The sulfuric acid was recovered and recycled by evaporating water, unreacted benzene and nitrobenzene by low pressure distillation. The heat integration was achieved by using the adiabatic heat for benzene recovery, and the pre-purification of nitrobenzene by distillation.

### 4 Nitration: from laboratory synthesis to process

Continuous flow nitration using miniaturized devices is an excellent approach to avoid issues related to heat transfer, mass transfer, homogeneity inside the reactor, and mixing. Large-scale continuous flow nitration was implemented in selected cases, mainly for basic organic substrates. At this stage, aromatic nitrations, whose process economics are significantly affected by the selectivity of specific isomers, are considered for the continuous flow approach. In addition to a few important issues, two parameters that help to tune the isomeric ratio of products will be discussed with the objective to illustrate the development from a synthesis procedure to an actual process.

#### 4.1 Nitrating agents used under continuous flow conditions

For industrially important aromatic nitrations the conventional nitrating mixture (40:60) and the mixture of sulfuric acid with fuming nitric acid are the nitrating agents of choice. The advantages of using the nitrating mixture as nitrating agent are known and the use of sulfuric acid in large quantity, which promotes the generation of nitronium ions and the absorption of the generated water is ideally suited for many cases. In Figure 1 an overview of the industrial relevance of nitrating agents is presented. About 30.5% of these nitrations specifically rely on the classical nitrating mixture as preferred nitrating agent. The trapping of water is crucial to avoid dilution and the crunch of nitronium ion. The neutralization of acids and the removal of salts formed during neutralization are costly and unavoidable steps if nitrations are carried out in large scale. The presence of sulfuric acid in large quantity reduces the actual production capacity from a given reactor. On the other hand, fuming nitric acid as nitrating agent allows circumventing the use of sulfuric acid and thereby simplifies neutralization and salt separation steps. However, it needs to be noted that the handling and the storage of fuming nitric acid is not safe. There are great future prospects for conducting aromatic nitration under continuous flow conditions. To foster nitration under continuous flow conditions, it is necessary to develop continuous (i) dilution, neutralization, extraction and salt separation steps while using the nitrating mixture and (ii) dilution, extraction and possibly enrichment of acid by evaporation and its recycling while using the fuming nitric acid. The amount of sulfuric acid and/or nitric acid strongly depends on the activity of the organic substrate. It is always useful to prepare the nitrating mixture with different compositions of HNO_3_ (fuming or concentrated) and H_2_SO_4_ inline before the mixture comes in contact with the organic substrate. Similarly, the use of different mol ratios of the nitrating agent with respect to the organic substrate can also be explored at laboratory-scale development. Such practices will save a significant amount of time for a given study and for exploring the parametric effect.

#### 4.2 Heat management in nitration

With respect to the heat management in continuous flow nitration, the approaches, which are found in the literature can be classified in two categories. The first approach uses the microreactors or flow reactors with built-in channels as depicted in Figures 3, 6, 9 or zones for heat transfer. i.e., integrated microreactors [38], where the heat transfer occurs from one or two faces of the microchannel with most of the channels having a square or rectangular cross-section. In the second approach the flow reactor is immersed inside a constant temperature bath as depicted in Figures 2, 4, 7 and 8. In this case, the constant temperature is guaranteed by the constantly circulated heat transfer fluid with an external temperature control. In general, the rate of heat removal strongly depends on the heat transfer area, the thermal conductivity of the device material, the superficial velocity of the heat transfer fluid, the superficial velocity of the reacting fluid, and the specific heat capacity of the reacting fluid. Thus, for the devices with build-in heat management system the actual area available for heat transfer is only half of the total reacting fluid wetting area. On the other hand, the immersed systems provide a complete exposure to the heat transfer fluid. Thus, identical heat transfer rates can be achieved with either a lower heat transfer fluid temperature or a lower flow rate of heat transfer fluid for the immersed microfluidic systems compared to the built-in channels. Generally, the immersed systems with external temperature control are less sophisticated and are therefore cheaper. The advantage of the integrated microreactors is in their compactness. Immersed microreactors are advantageous due to their simplicity and smaller number of connections. In practice, the nature of heat transfer remains more or less the same. The modularity of the entire system and the utility costs are essential factors for choosing which heating mode to choose for scale-up.

#### 4.3 Interfacial reactions: real and apparent kinetics

The nitration of organic substrates using different nitrating agents can be classified based on whether the reaction is homogeneous or heterogeneous (multiphase). Taking into account the liquid phase substrates alone, almost 78% of the reactions reported in the literature, are two-phase reactions based on an analysis similar to the one depicted in Figure 1. In such systems the reaction takes place only after one of the reactants diffuses into the other. Thus, the rate of mass transfer controls the reaction rates. As a result, the actual reaction kinetics reported in the literature does not always explicitly indicate whether the mass transfer limitation was overcome. While this limitation actually renders much of the data from the literature on conversion and selectivity from experiments using round bottom flask or stirred systems useful only in terms of the products, it cannot be directly utilized for the estimation of kinetic parameters of experiments under different conditions. The Taylor flow or dispersion achieved in miniaturized systems significantly reduces the mass transfer limitations. To achieve reliable data, the flow rates should be adjusted in a way that avoids very long slugs. Although most of the regime maps in the literature are subject to the physical properties of the fluids as well as the channel dimensions and shape, an approximate analysis of flow regimes may be useful to ensure that the mass transfer limitations are overcome.

#### 4.4 Handling of solids

A large proportion of either the organic substrates or their nitro derivatives reported in the literature are solids. In the conventional method the nitration of solid substrates is facilitated by using either a solvent in large excess – typically a weak acid, which does not get nitrated such as acetic acid – or sulfuric acid. The latter is mainly used for deactivated substrates to not only facilitate the dissolution of the substrate, but also form a complex, which increases the activity of the substrate for getting nitrated. On the other hand, a solid product precipitating during the reaction, results in a significant increase in the viscosity of the solution. While the use of techniques like reactions under sonication to prevent wall adhesion or particle agglomeration, inducing mechanical vibrations to the system to keep the solids in suspension, and the usage of high flow rates to ensure that the superficial flow velocity is higher than the settling velocity of solids is feasible, an optimal combination of using solvents in adequate quantity and maintaining a high superficial velocity are best suited to prevent precipiation. However, given a complex geometry of a microreactor the low pressure zones inherently enhance the possibility of particle accumulation in these zones even at high velocities. In laboratory equipment the simplest concept to avoid clogging relies on dilution.

#### 4.5 Reliability of the analysis and establishing mass balance

For most nitrations, the products and the reactants are very poorly soluble in the aqueous phase, which comprises diluted nitric acid or diluted spent acid. In most of the cases, although the outlet product mixture is extracted by using a common organic solvent such as toluene, ethylene dichloride, hexane, ether, a certain fraction of the mono and dinitro derivatives and the reactant remain the aqueous phase. Consequently, an analysis based on the organic phase alone may not result in conclusive information about the extent of the reaction. Furthermore, ignoring the composition of the aqueous phase leads to an inaccurate measurement of the extent of byproducts. These points become important when the reactant has certain, even very small impurities, which may get nitrated rapidly and remain dissolved in the highly polar acidic medium. In most cases, publications about continuous flow nitration do not indicate whether the aqueous phase was also analyzed. Moreover, it is essential to establish the exact mass balance for a given reaction, so that the efficacy of the process can be evaluated.

### 5 Conclusion

The analysis of the literature clearly indicates that it is feasible for anyone to setup a flow reaction system for the nitration of aromatic substrates. Flow setups allow for the rapid screening and optimization of parameters to achieve optimal conditions for the nitration of arenes. In most cases, the reaction needs to be terminated by inline hydrolysis. Semi-batch hydrolysis should be avoided, as the dilution of excess acid does not occur uniformly. This can pose a problem at a later stage because of the high concentration of the nitrated product in the presence of an excess of the nitrating agent. It is quite common that either the reactant or the nitro derivative may have limited solubility in the reaction mixture, which poses a significant challenge in ensuring that the flow synthesis can operate continuously.

Depending upon its density the precipitate generated during the reaction may respond differently to the flow conditions and the geometry of the channels. One of the ways to overcome the situation is by using a solvent [58] with a good solubility for the organic substrates/products at the cost of increasing the volume of the reactor. Furthermore, an additional purification step, typically distillation, is required. While these procedures are routine on a laboratory scale they may not be suitable for large-scale manufacturing. In such cases, it is possible to avoid the need of an additional reagent or dilution with a substrate (if in liquid phase) that serves as the solvent.

In practice, nitrations are followed by a reduction step to end up with an amino group. Commonly, nitrations yield several products as discussed in Schemes 4, 6, 8 and 14, which impedes direct hydrogenation. Thus, the separation of isomers after the nitration is an essential step. It will always remain a challenge to develop a liquid phase nitration that selectively yields only one nitro isomer or to achieve a separation protocol for the mixtures of reduction products, namely for different amines. Due to safety issues fuming nitric acid was only rarely used under batch conditions. However, fuming nitric acid becomes an option with the continuous flow approach, as safety issues can be better handled. This approach will also help to estimate the kinetic parameters for nitration in a more accurate manner, so that it is possible to have a good control on the temperature inside the reactor.

Now that the continuous flow nitration has emerged as an established technique that helps to control the yield of the desired isomer to some extent, it is necessary for the chemists and chemical engineers to work together for optimizing single protocols based on detailed mathematical analysis. The key is to understand the rate-controlling step, which can be interfacial mass transfer, kinetics or even thermodynamics as in the case of a solubility limited reaction.
